# Neuronal and Astroglial Correlates Underlying Spatiotemporal Intrinsic Optical Signal in the Rat Hippocampal Slice

**DOI:** 10.1371/journal.pone.0057694

**Published:** 2013-03-01

**Authors:** Ildikó Pál, Gabriella Nyitrai, Julianna Kardos, László Héja

**Affiliations:** Department of Functional Pharmacology, Institute of Molecular Pharmacology, Research Centre for Natural Sciences, Hungarian Academy of Sciences, Budapest, Hungary; Charité University Medicine Berlin, Germany

## Abstract

Widely used for mapping afferent activated brain areas *in vivo*, the label-free intrinsic optical signal (IOS) is mainly ascribed to blood volume changes subsequent to glial glutamate uptake. By contrast, IOS imaged *in vitro* is generally attributed to neuronal and glial cell swelling, however the relative contribution of different cell types and molecular players remained largely unknown. We characterized IOS to Schaffer collateral stimulation in the rat hippocampal slice using a 464-element photodiode-array device that enables IOS monitoring at 0.6 ms time-resolution in combination with simultaneous field potential recordings. We used brief half-maximal stimuli by applying a medium intensity 50 Volt-stimulus train within 50 ms (20 Hz). IOS was primarily observed in the *str. pyramidale* and proximal region of the *str. radiatum* of the hippocampus. It was eliminated by tetrodotoxin blockade of voltage-gated Na^+^ channels and was significantly enhanced by suppressing inhibitory signaling with gamma-aminobutyric acid(A) receptor antagonist picrotoxin. We found that IOS was predominantly initiated by postsynaptic Glu receptor activation and progressed by the activation of astroglial Glu transporters and Mg^2+^-independent astroglial N-methyl-D-aspartate receptors. Under control conditions, role for neuronal K^+^/Cl^−^ cotransporter KCC2, but not for glial Na^+^/K^+^/Cl^−^ cotransporter NKCC1 was observed. Slight enhancement and inhibition of IOS through non-specific Cl^−^ and volume-regulated anion channels, respectively, were also depicted. High-frequency IOS imaging, evoked by brief afferent stimulation in brain slices provide a new paradigm for studying mechanisms underlying IOS genesis. Major players disclosed this way imply that spatiotemporal IOS reflects glutamat*ergic* neuronal activation and astroglial response, as observed within the hippocampus. Our model may help to better interpret *in vivo* IOS and support diagnosis in the future.

## Introduction

Reflecting genuine excitability of neural cells, the label-free intrinsic optical signal (IOS) [1], [2] can essentially be detected at cellular resolution both *in vivo* and *in vitro* [3], [4], [5], [6], [7]. IOS is widely used for non-invasive imaging of spatiotemporal neural activity of patients in intra-operative neurosurgery [8], [9], [10]. Paradoxically, despite the diagnostic power of IOS imaging technique, detailed understanding of molecular and cellular processes underlying the generation of afferent evoked spatiotemporal IOS is still lacking [11], inviting to study the relationship between synaptic activity and the IOS signal.

In vitro IOS in isolated brain slices is presumed to be almost exclusively caused by changes in light absorption and light scattering [1], [2]. Light absorption changes are mostly attributed to the intrinsic cytochromes like mitochondrial porphyrins and residual hemoglobin [12]. Excitoxicity related light scattering changes are associated with dendritic beading [13], while spreading depression related IOS is ascribed to changes in mitochondrial architecture or metabolic activity [1], [2], [14], [15]. Local changes in light scattering due to activity-dependent cell swelling [1], [16] and alterations of the extracellular volume [17], [18] are regarded as the principal component of the osmotic pressure induced and afferent stimulation evoked IOS [1], [19].

In order to better understand the mechanistic clues, afferent evoked IOS generation has been studied *in vitro* in various brain slice preparations [1], [20], [21], [22]. Afferent stimulation evoked IOS was seen to be dependent on postsynaptic activity in hippocampal [7] and neocortical slices [20], [23]. In neocortical slices IOS has been found to more sensitively reflect neuronal excitatory activation than postsynaptic activity [20], [24], however the detailed molecular dissection of the contribution of excitatory neurotransmitter receptors is still missing.

The proposal that afferent evoked IOS is attributed to neuronal activity induced cell swelling is based on the fact that it was found to be strongly dependent on extracellular [Cl^−^] [7], [18], addressing the contribution of anion channels and transporters. The role for glial glutamate uptake in cell swelling has also been shown [25], [26]. Reportedly, astrocytes generate odor evoked *in vivo* IOS via modulation of cerebral blood flow, while sensory organ stimulation evoked neural activity is also coupled to astrocytes through glial glutamate uptake [27]. These findings conclusively suggest that it may be the glial uptake of glutamate that couple neuronal activity to IOS.

To better understand the molecular and cellular processes of IOS generation we applied fast imaging of brief Schaffer collateral stimulation evoked IOS in hippocampal slices with simultaneous local field potential recordings. Numerous targets possibly influencing IOS generation (voltage-gated Na^+^ channel, gamma-aminobutyrate A receptor, neuronal and astroglial Glu receptors, major astroglial Glu transporter, neuronal K^+^/Cl^−^ cotransporter KCC2, Na^+^/K^+^/Cl^−^ cotransporter NKCC1, non-specific Cl^−^ channels, volume-regulated anion channel, VRAC) were tested by their inhibitors tetrodotoxin, picrotoxin, 6-cyano-7-nitroquinoxaline-2,3-dione (CNQX) and/or DL-2-amino-5-phosphonopentanoic acid (APV), dihyrokainic acid (DHK), furosemide, bumetadine, 4,4′-diisothiocyanatostilbene-2,2′-disulfonic acid DIDS, 4-(2-butyl-6,7-dichloro-2-cyclopentylindan-1-on-5-yl)oxybutyric acid (DCPIB), respectively.

## Materials and Methods

### Ethics Statement

Animals were kept and used in accordance with the European Council Directive of 24 November 1986 (86/609/EEC), the Hungarian Animal Act, 1998. All experiments involving animals were done by the approval of the Animal Testing Committee of the Research Centre for Natural Sciences, Hungarian Academy of Sciences and by the approval of the Ministry of Agriculture and Rural Development, Hungary. All efforts were made to reduce animal suffering and the number of animals used.

### Chemicals

Picrotoxin, DIDS, DCPIB and furosemide were purchased from Sigma-Aldrich Co. (St. Lois, MO, USA). Tetrodotoxin (TTX), DHK and bumetanide were purchased from Tocris Bioscience (Bristol, UK). CNQX and APV were purchased from Abcam Biochemicals (Cambridge, UK).

### Brain Tissue Slices

Transverse 400 µm thick hippocampal-entorhinal cortex slices were cut by a vibratome (Leica VT1000S, Leica Microsystems, Wetzlar, Germany) from male Wistar rats (Toxicoop, Budapest, Hungary) as described elsewhere [28]. P11–20 animals were used for simultaneous field potential and single cell recordings and P21–50 animals were used for simultaneous field potential and IOS recordings. Slices were put in a submerged type recording chamber and perfused by carbogen gas-saturated artificial cerebrospinal fluid (ACSF, in mM: 129 NaCl, 10 glucose, 3 KCl, 1.25 NaH_2_PO_4_, 1.8 MgSO_4_, 2 CaCl_2_, 21 NaHCO_3_, pH 7.4, 36°C).

### Electrical Stimulation

Stimulating electrode was placed in the trajectory of the Schaffer collaterals, in the *str. radiatum* of the CA3 region and field responses were recorded from the CA1 pyramidal layer. When two recording electrodes were used, the second electrode was placed in the CA1 *str. radiatum*. In the text these measurements are referred as two recording electrode measurements.

Single square wave pulses of 0.2 ms duration were delivered through a bipolar tungsten electrode (75 µm diameter, Microprobes for Life Science). Stimulation intensity was set to evoke half maximal field response (40–50 V). Stimulus train (10 stimuli/20 Hz) or single pulses were applied for IOS and single cell measurements, respectively. Each stimulus was preceded by a 200 ms long delay period for baseline calculations. Three trials were taken in every 10 and 5 min for IOS and single cell measurements respectively.

### Field Potential and Single Cell Recording

Extracellular (2–5 MΩ) and cell-attached (5–9 MΩ) electrodes were filled with ACSF. For cell-attached recordings, holding potential was set to 0 mV. To perform whole-cell current-clamp recordings 3–5 MΩ pipettes were filled with intracellular solution (containing in mM: 135 KGlu, 10 NaCl, 0.05 CaCl_2_, 2 adenosine-5′-triphosphate (ATP; Mg^2+^ salt), 10 4-(2-hydroxyethyl)-1-piperazineethanesulfonic acid (HEPES), 3 ethylene glycol-bis(2-aminoethylether)-N,N,N′,N′-tetraacetic acid (EGTA); pH set to 7.3 [29]. The tip of the patch electrode was placed in the 100 µm range of the extracellular electrode. In simultaneous field potential and single cell recording three trials were taken in every 5 min to minimise the damage to the patched cell. Recordings were performed with a two-channel Multiclamp 700A amplifier (Axon Instruments, Foster City, CA, USA). Traces were low-pass filtered at 2 kHz and digitized at 10 kHz with a Digidata 1320A A/D board (Axon Instruments, Foster City, CA) controlled by a computer running pClamp8 (Axon Instruments, Foster City, CA, USA).

### Optical Recording

Slices were illuminated with a halogen lamp source (maximum intensity: 100 W) passed through a band-pass filter (700±40 nm, Chroma Technology, Rockingham, VT). The transmitted light was collected with a Wutech H-469IV photodiode array that is part of the Redshirt Imaging integrated Neuroplex II imaging system, mounted on the front port of an Olympus BX51WI microscope (1.6 kHz sampling frequency, 500× amplification, 0.5 Hz high-pass filtered [30]). Each diode corresponds to an approximately 70×70 µm square area. The data were acquired and displayed with Neuroplex software. Three trials were taken in every 10 min.

### Drug Application

Drugs were superfused (3–4 ml/min) for 15 min after at least two control trials have been recorded. For evaluating drug effects, two trials were taken after 5 and 15 min of drug application. If the effect of the drug in the two sampling point were not significantly different the average of the two points was used for further evaluation. 5 µM TTX was used to ensure the blockade of all sodium channel mediated current [31].

### Elevated [K^+^] Evoked IOS

Elevated [K^+^] buffer was made by the substitution of 47 mM NaCl of the ACSF to have final [K^+^] of 50 mM. ACSF with [K^+^] = 50 mM was pressure-injected to the surface of the slice through a patch pipette (5–10 µm diameter) for 10 seconds. The pipette was placed 100 µm above the surface of the slice to avoid pressure induce artifacts. As a control, normal ACSF was administered in the same manner at the same position. Elevated [K^+^] puff was applied in the CA1 *str. oriens* and *str. radiatum* for 10 seconds following a 10 second long control period. IOS was monitored for 1 minute with the same illumination and adjustments as used for the afferent stimulation evoked IOS.

### Primary Data Processing and Data Analysis

To evaluate the electrophysiological signal the slope of the field excitatory postsynaptic potential (fEPSP) was measured for each field response. For field responses measured in the pyramidal layer the amplitude of the population spike (PS) was also calculated. Field response parameters were calculated according to Anderson et al. [32]. The population spike amplitude was measured as the amplitude from the PS onset to the intersection with a tangent line drawn between the PS onset and the PS offset.

Since we used 10 stimuli to evoke IOS, the sum of the fEPSP slope or PS amplitude of the 10 responses were calculated and compared to IOS parameters.

The spreading velocity of neuronal activation was estimated by measuring the delay of the population spike peak from the stimulation artifact in the single electrode measurements or the delay between the PS peaks of the two the recording electrode in the two electrode measurements. The estimated values did not differ for the two calculation methods therefore the values were averaged.

In the IOS measurements three trials were averaged to reduce the noise. IOS signals were accepted if the signal to noise ratio has exceeded 5. Five parameters of the IOS curves were calculated: amplitude (average of the baseline recorded before the stimulus was subtracted from the maxima), location of the maxima, length of the signal (calculated from the signal start to the point where the signal felt to 5% of the amplitude), 10–90 slope and 90-10 decay. The number of diodes with signal/noise >5 was also determined. The integrative response of the whole slice was characterized by the sum of IOS amplitudes measured simultaneously on all diodes (referred to as IOS sum. ampl.). In addition, the IOS curve shape was featured by the average slope and decay parameters calculated by sum of all diodes slope or decay divided by the number of diodes with signal/noise >5 (referred to as IOS slope and IOS decay, respectively). Sum of all IOS parameters have also been calculated for the sole CA1 and CA3 (Figure S1) but, except in the case of DHK, the effects of the drugs were not significantly different when compared to the sum of the whole slice. Therefore, the field potential parameters were compared to the sum of IOS parameters of the whole slice to increase the signal/noise ratio of the IOS measurements. The difference of DHK effect in the CA1 and CA3 is caused by the fact that pyramidal layer in the CA3 was not always in the field of view in these measurements.

To visualize the spatial pattern of changes due to drug application differential images were calculated. For each diode the IOS difference during and before drug application was calculated (referred to as ΔT/Tc where ΔT is the difference in transmittance and Tc is the average value of the control trials) and illustrated by the appropriate color of the colorbar.

Spreading velocity of the IOS was estimated by dividing the distance between two diodes by the delay between the peaks of the first derivative of the two IOS signals.

Mann-Whitney U test was used for statistical testing with P<0.05 considered significant and data are reported as means ± SEM. Data processing, analysis, and graphical representations were performed with pClamp9 (Axon Instruments), Origin 8.0 (Origin-Lab) softwares and custom written scripts in MATLAB 6.5 (MathWorks, Nattick, MA) environment.

## Results

### Afferent Stimulation Evoked IOS in Rat Hippocampal Slice

**Figure 1:**
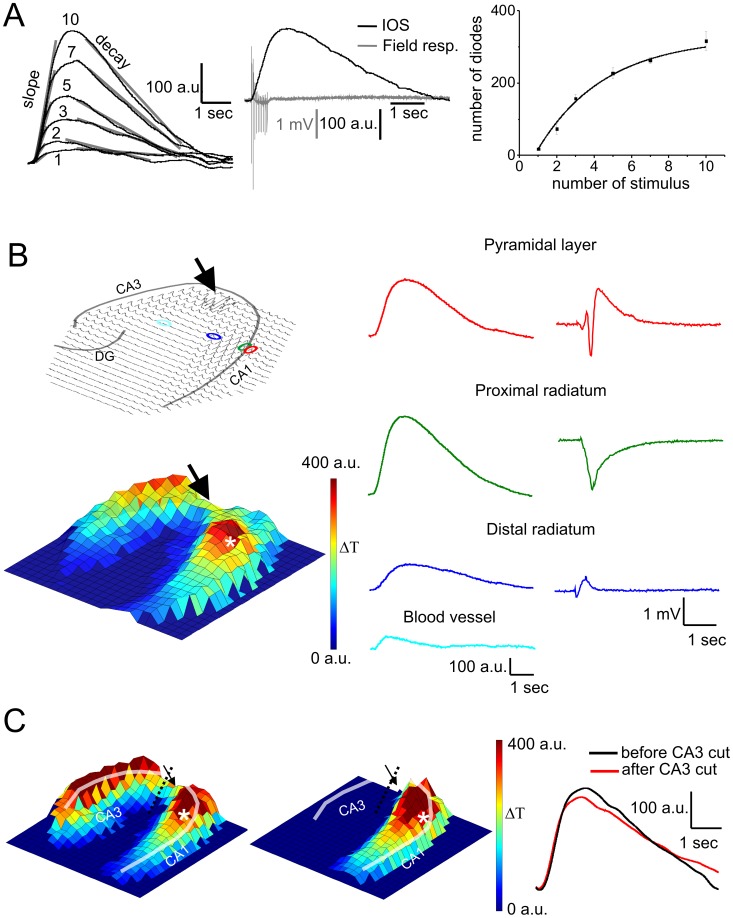
Time course, stimulation dependency and regional distribution of IOS and field response. **A left:** Changes of IOS shape and amplitude with the increasing number of stimuli. Numbers above that curves indicate the stimulus number. Transparent lines show the fitted linear function to calculate the slope and decay of the IOS curves. **A middle:** Time course of the IOS and field response **A right:** Correlation between the number of diodes containing IOS and the stimulus number (R^2^ = 0.99). **B Top left:** Overview of the IOS signal on the 464 element photodiode array. Transparent lines indicate the pyramidal cell layer and the granular cell layer of the *dentate gyrus* (DG). The position of the stimulating electrode is marked by an arrow and the sites of field potential recording electrodes are indicated by colored elipses. **B Bottom left:** 3D representation of the IOS amplitude. The position of the stimulating and recording electrodes are marked by an arrow and an asteriks, respectively. The colorbar indicates the transmittance change compared to the resting light intensity. **B Right:** Representative IOS and field response traces recorded at different locations of the slice. The exact location of the traces are indicated by the coloured elipses on the Top Left image. **C Left and Middle:** 3D IOS amplitude maps before (left) and after (middle) making a cut between the stimulating electrode and CA3. Transparent line indicates the pyramidal layer. The position of the stimulating electrode is marked by an arrow and the site of field potential recording is indicated by an asterisk. **C Right:** Representative IOS traces near the recording electrode (marked by white asterisk) before (black) and after (red) making a cut between the stimulating electrode and CA3. The slight difference between the curves is due to the slightly decreased electrophysiological characteristics after making cut.

For IOS measurements we used a 464 element photodiode-array (PDA) detector instead of the commonly applied charge-coupled device (CCD) camera. The PDA detector enables IOS detection with 0.6 ms time resolution, making it achievable to align the optical signal with the simultaneously measured electrophysiological recordings. To quantify the effect of the applied inhibitors, we calculated six different parameters for each IOS curves: amplitude, location of the maxima, length, 10–90 slope, 90-10 decay and the number of diodes showing significant IOS activity (Figure 1A, B). Amplitude of the IOS signal in the close vicinity of the electrophysiological recording site as well as summa amplitude measured on all diodes covering the entire hippocampus were compared to electrophysiological signal parameters, namely the amplitude of the population spike (PS) and the slope of the field excitatory postsynaptic potential (fEPSP). The amplitude of the population spike measures the synchronous firing of the neighboring neurons [33], while the slope of the fEPSP measures the currents generated by the synaptic activation of pyramidal cells [34].

Hippocampal IOS was evoked by applying brief stimuli to the Schaffer collaterals. The stimulation protocol was optimized by varying the stimulus number to find the lowest stimulus intensity that generates measurable IOS. One or two stimulations were sufficient to generate detectable IOS, albeit with low signal to noise ratio (1.5). Ten stimuli during 450 ms at 20 Hz stimulation frequency were needed to detect reliable IOS (Figure 1A). Our protocol therefore applies at least four times fewer and eight times shorter stimuli than the stimulus protocol used in previous studies [7], [35]. IOS amplitudes measured in the close vicinity of the recording electrode in the pyramidal layer were found to be linearly correlating to the stimulus number and also to the PS amplitude and fEPSP slope (R = 0.98 and R = 0.99, respectively), indicating that *in vitro* manifest IOS resides in neuronal activity. It is noteworthy to mention that the slope (R = 0.99) and decay (R = 0.97) of IOS were also changing with the stimulus number (Figure 1A). Under control conditions, IOS measured close to the recording electrode started at the stimulation onset, reached its maxima within 2.5±0.03 s and fell back to the baseline in the next 3.5 s (Figure 1A). Due to the brief stimulation protocol, the IOS reached its peak at least two times earlier and declines seven times faster than in previous studies [7], [35].

Occasionally, we observed small-amplitude IOS signals with short latency and duration in the *str. radiatum* and *lacunosum-moleculare*. Insensitivity of these signals to TTX suggests that they are attributed to the contraction of medium size blood vessels initiated by the activation of voltage-gated calcium channels [36] in the vicinity of the stimulating electrode. Vessel associated signals were distinguishable by their early maxima (<1.5 sec), short duration (<2 sec), low slope and decay (Figure 1B Right) and were subtracted from the local IOS when occurred.

IOS was found to be most prominent in the proximal *str. radiatum* and *str. pyramidale* of the hippocampus, showing the highest amplitude signals in the CA1 region, corresponding to the innervated area of the stimulated Schaffer collaterals (Figure 1B). IOS was also detected in the CA3 region of the pyramidal layer most probably due to the activation of the CA3 to CA3 associational projections and/or the activation of the mossy fibers. The IOS amplitude gradually declined along the CA1 *str. radiatum* (23.9±1.8%/100 µm) from the proximal to the distal regions. Since most of the glial cells are located in the distal region of the *str. radiatum*, the high IOS amplitude in the *str. pyramidale* in our experiments clearly conflicts with previously suggested predominant glial origin [7] and indicates that neuronal activity also contribute to IOS.

In order to correlate the IOS amplitude and the electrophysiological activity in the *str. radiatum* and *str. pyramidale* layers of the CA1 region of the hippocampus, we simultaneously measured the field response with two recording electrodes. One of the extracellular recording electrodes was placed in the CA1 pyramidal layer close to the stimulating electrode as the reference, while the other electrode was placed *i)* in the pyramidal layer at least 300 µm from the stimulating electrode or *ii)* in the proximal and distal *str. radiatum* (Figure 1B Right). The shape and amplitude of the IOS and field response were found to be characteristically varying with the distance from the pyramidal layer (Figure 1B Right).

First, fEPSP slopes measured on recording electrodes in different positions (*str. pyramidale*, distal and proximal *str. radiatum*, Figure 1B) were compared to the IOS amplitude measured near to the recording electrodes. Because PS is not visible at all the measured locations, absolute value of the fEPSP slope was compared to IOS amplitude. The spatial pattern of the IOS signal does not correlate precisely with the absolute value of the fEPSP slope. The lack of the correlation is due to the high IOS amplitudes and low fEPSP slope values, especially in the distal *str. radiatum* indicating that glial cells in this area probably contribute more to the IOS than to the fEPSP slope. In the pyramidal layer IOS amplitude and fEPSP slope were linearly correlated (R = 0.86), suggesting that in the pyramidal layer the contribution of neurons to IOS is in line with their contribution to fEPSP slope. Similarly, the existence of correlation in the pyramidal layer suggests that neurons contribute to both the IOS signal and the fEPSP slope.

The spatial pattern of IOS indicates that CA3 region is also activated possibly via the axonal backpropagation of the stimulus. Activation of CA3 pyramidal cell can reverberate to the CA1 through the hippocampal-entorhinal loop [37] which can also contribute to the IOS signal measured in the CA1. In order to clarify the contribution of CA3 network activity to IOS time course and spatial distribution in the CA1 region, IOS signals in CA1 before and after making a cut between the stimulation electrode and CA3 were compared. After physically decoupling the CA3 and CA1 activity IOS completely disappeared from the CA3 region (N = 4, Figure 1C). The spatial pattern and time course of IOS in the CA1, however, remained unchanged suggesting that Schaffer collateral stimulation evoked neuronal activity is the only source of IOS in the CA1.

Sum of IOS amplitude on all diodes correlates with the PS amplitude (R = 0.72). The fact that the PS amplitude measured at a single location and the summa IOS amplitude of the slice correlates suggests that both the IOS signal and the field response represent the general excitation level of the slice.

### Temporal Dissection of IOS Generation

**Figure 2:**
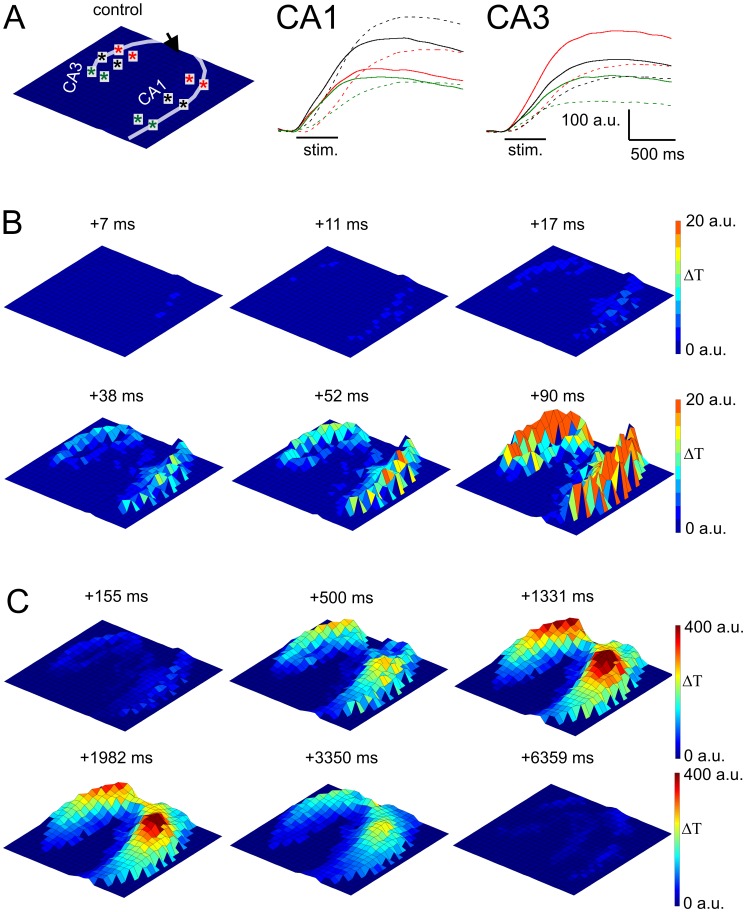
Temporal dissection of the IOS signal. **A Left:** Orientation of the slice. Transparent line indicates the pyramidal layer and the arrow indicates the position of the stimulating electrode. **A Middle and Right:** Representative IOS traces from the CA1 and CA3 region respectively. The origins of the traces are indicated by colored asterisks on **A left.** Dashed lines indicate IOS traces originating from the *str. radiatum*. **B:** 3D IOS amplitude maps at different time points showing the spatial appearance of IOS in the first 90 ms following stimulus onset. The orientation of the slice is represented on **A left. C:** 3D IOS amplitude maps at different time points showing the spatial dynamics of IOS at a longer time-scale. The orientation of the slice is represented on **A left.** The colorbar indicates the transmittance change compared to the resting light intensity.

The high-frequency sampling rate of the PDA device allowed us to temporally dissect the IOS generation process (Figure 2, Video S1 and Video S2). Following the stimulation, IOS appears first in the CA1 *str. pyramidale* at Schaffer collateral end points than in the *str. radiatum* and other parts of the *str. pyramidale* of the CA1 and CA3. This phenomenon implies that neuronal activation precedes IOS generation, probably because IOS exceeds detection limit in the somatic region of pyramidal cells faster than in the dendritic region or neuronal swelling might arise faster than glial swelling.

In the CA1 *str. radiatum*, IOS initially appears at sites where Schaffer collaterals form en passant synapses on CA1 pyramidal neurons [38] than it propagates in two directions: 1) toward the stimulation electrode and 2) toward the *subiculum* (Figure 2, Video S1 and Video S2). The estimated spreading velocity of the IOS signal is 0.23±0.05 µm/ms that is two order of magnitude slower than the spreading velocity of the field potential signal (240±12 µm/ms), but one order of magnitude faster than the spreading velocity of the ATP-mediated astrocytic Ca^2+^ waves (0.014±0.005 µm/ms) induced by electrical stimulation measured in mice neurocortex [39]. The spreading phenomenon of IOS in the CA1 *str. radiatum* can be explained by activation of the neighboring astrocytes through gap junctions or through glia-to-glia signaling [40]. In contrast to CA1, IOS in the CA3 spreads only toward the neighboring *str. radiatum*, but not along the *str. pyramidale*.

The appearance of IOS in the CA3 is most probably due to the antidromic activation of CA3 to CA3 associational projections. It is to note that IOS quickly appears in the distal CA3 radiatum, most likely indicating the activation of the mossy fiber.

IOS dynamics in the CA1 and CA3 are also significantly different (Figure 2A Middle and Left). In the CA3 that is stimulated antidromically, IOS traces in the *str. radiatum* are always smaller than in the pyramidal layer, while in the orthodromically activated CA1 IOS traces in the *str. radiatum* overshoot those recorded in the *str. pyramidale*. These delicate differences indicate that with high frequency IOS imaging not only the spreading pattern of excitation can be monitored, but activation patterns due to the different synaptic arrangements can also be discriminated.

### IOS is the Consequence of Neuronal Activation

To further characterize the relationship between IOS and components of the field response (fEPSP slope and PS amplitude) we compared changes of the field response components to summa IOS amplitude during different drug applications.

The stability of IOS and field potential baselines were tested for the duration (50 min) of the pharmacological experiments. Despite a slight positive trend in all the calculated parameters (end values for PS amplitude 101±1% of control, for summa IOS amplitude 102±2% of control, for fEPSP slope 103±2% of control), no significant changes were observed during the experiments. Drug effects were considered significant if the changes exceeded two times the baseline change.

**Figure 3:**
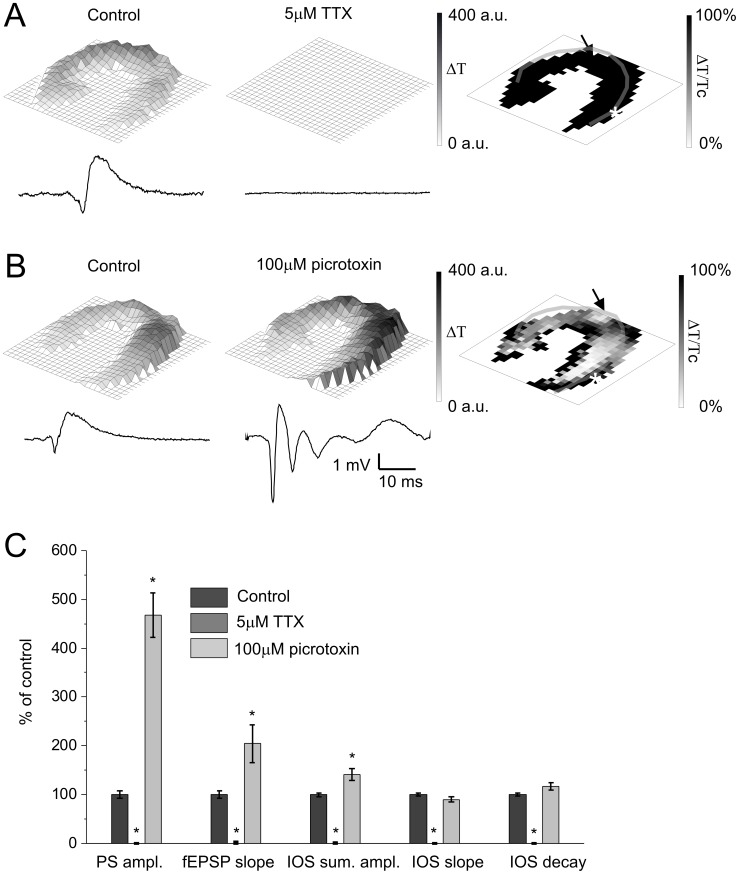
IOS amplitude is directly dependent on neural activity. **A Left and Middle:** Representative IOS amplitude map and field response curve under control condition and 5 µM TTX application. The colorbar indicates the maximum change of the transmittance compared to the resting light intensity. **A Right:** Spatial visualization of the percentage of control changes of IOS signal caused by TTX application. **B Left and Middle:** Representative IOS amplitude map and field response curve under control condition and 100 µM picrotoxin application, respectively. The colorbar indicates the maximum change of the transmittance compared to the resting light intensity. **B Right:** Spatial visualization of the percentage of control changes of IOS signal caused by 100 µM picrotoxin application. **C:** The effect of 100 µM picrotoxin and 5 µM TTX on the field response and IOS parameters in percentage of control. Asterisks indicate significant changes compared to control (P<0.05 Mann-Whitney U test, N = 5). Transparent lines on panel A, B right indicate the pyramidal cell layer. The position of the stimulating and recording electrode are marked by an arrow and asteriks, respectively.

Blockade of voltage-gated sodium channels by TTX (5 µM) completely eliminated both the field response (PS amplitude and fEPSP slope: 0±3 and 0±0% of control, respectively; N = 5) and IOS (IOS sum. ampl.: 0±1% of control, N = 5). IOS completely disappeared from all cell layers (Figure 3A), demonstrating that the passive voltage propagation in the tissue is insufficient to evoke IOS, therefore neuronal activation is necessary for IOS generation.

Intensification of neuronal activity by applying the GABA_A_ receptor antagonist picrotoxin (100 µM) enhanced both IOS (IOS sum. ampl.: 141±12% of control, N = 5) and field response (PS amplitude and fEPSP slope: 468±46 and 204±39% of control, respectively; N = 5, Figure 3B and C). It is to note that the increase in IOS amplitude (41±12% of control) much more resembles the increase of fEPSP slope (104±39% of control) than that of PS amplitude (368±46% of control), suggesting that IOS more sensitively reflects the changes in the synaptic currents than the neuronal firing. Interestingly, the most pronounced changes in IOS amplitude were observed in the pyramidal layer and in the distal *str. radiatum* (Figure 3B). IOS changes in the distal *str. radiatum* are probably caused by the extension of the excited area, while IOS enhancement in the pyramidal layer corresponds to the high expression level of the GABA*ergic* synapses on the soma and apical dendrites of pyramidal neurons [41]. These findings conclusively suggest that IOS is directly dependent on neural activity.

### Contribution of Neuronal and Astroglial Glutamate Receptor Activation to Afferent Activation Evoked IOS

**Figure 4:**
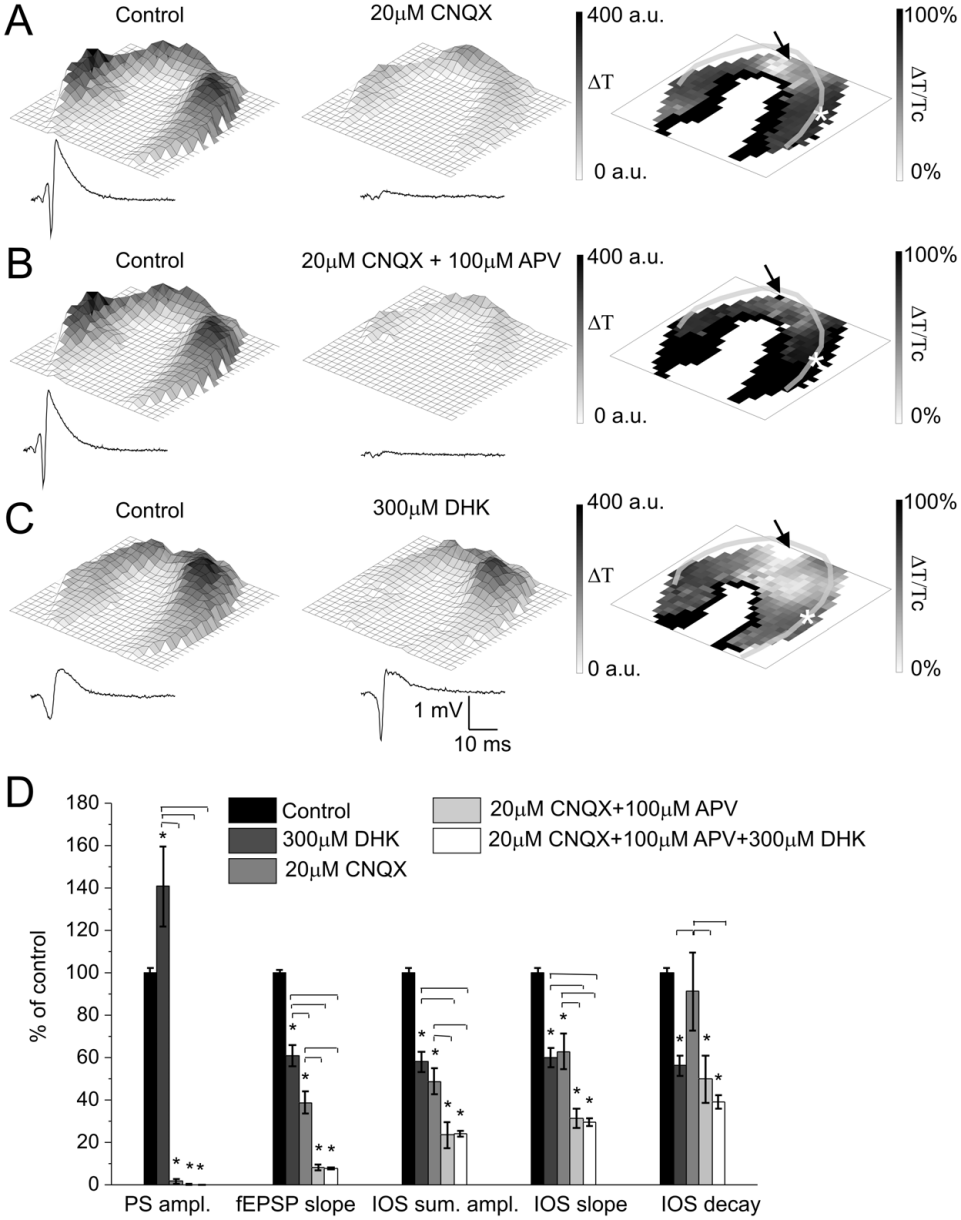
Ionotropic glutamate receptors and glial glutamate uptake plays a role in IOS. **A Left and Middle:** Representative IOS amplitude map and field response curve under control condition and under application of 20 µM CNQX, respectively. The colorbar indicates the maximum change of the transmittance compared to the resting light intensity. **A Right:** Spatial visualization of the percentage of control changes of IOS signal caused by CNQX application. **B Left and Middle:** Representative IOS amplitude map and field response curve under control condition and under application of 20 µM CNQX in combination with 100 µM APV, respectively. The colorbar indicates the maximum change of the transmittance compared to the resting light intensity. **B Right:** Spatial visualization of the percentage of control changes of IOS signal caused by APV and CNQX application. **C Left and Middle:** Representative IOS amplitude map and field response curve under control condition and 300 µM DHK application, respectively. The colorbar indicates the maximum change of the transmittance compared to the resting light intensity. **C Right:** Spatial visualization of the percentage of control changes of IOS signal caused by DHK application. **D:** Effect of 300 µM DHK and 20 µM CNQX alone and 20 µM CNQX in combination with 100 µM APV or 100 µM APV and 300 µM DHK on the field response and IOS parameters in percentage of control. Asterisks indicate significant changes compared to control (P<0.05 Mann-Whitney U test, N = 5−7) and horizontal bars above the columns denotes significant changes between columns (P<0.05 One-way ANOVA, N = 5−7). Transparent lines on panel A, B C right indicate the pyramidal cell layer. The position of the stimulating and recording electrode are marked by an arrow and asteriks, respectively.

2-amino-3-(5-methyl-3-oxo-1,2-oxazol-4-yl)propanoic acid (AMPA)/kainate receptor inhibition by CNQX (20 µM) decreased the PS amplitude (by 98±1% of control) and the fEPSP slope (by 61±5% of control) more robustly than the summa amplitude of IOS (by 51±6% of control, N = 6; Figure 4A and D). IOS decrease by CNQX was most prominent at the region innervated by the stimulated Schaffer collaterals (Figure 4A). It is to note that CNQX significantly decreased the slope of the average IOS curve (by 37±8% of control), but did not affect the decay phase, implying that CNQX selectively acts upon the IOS generation process and leaving the IOS terminating process intact.

Subsequent addition of the NMDA receptor antagonist APV (100 µM) further decreased the remaining summa IOS amplitude (by 25±6% of control, N = 5) mainly in the CA1 *str. radiatum* far from the stimulating electrode (Figure 4B). APV also decreased the fEPSP slope (by 31±1% of control), but did not affect the PS amplitude (Figure 4D). Both IOS slope (by 79±5% of control) and decay (by 50±11% of control) decreased but to different extents. This finding suggests that the dominant component (77%) of the IOS is dependent on activation of ionotropic Glu receptors after stimulation of Shaffer collaterals.

Since the removal of the Mg^2+^ blockade from NMDA receptors usually requires AMPA receptor activation [42], [43], [44] and glial NMDA receptors are only weakly sensitive to Mg^2+^ block [42], [43], the additional inhibitory effect of APV suggests the substantial involvement of Mg^2+^-independent glial NMDA receptors [42], [43]. IOS, however, could not be completely blocked (23±6% of control remained) by application of both CNQX and APV. This residual IOS may correspond to Glu uptake initiated by presynaptic glutamate release or could be the result of antidromic neural stimulation as observed by Andrew and MacVicar [45].

### Contribution of Astroglial Glu uptake to Afferent Activated IOS in the Hippocampal Slice

To further explore the role of glial Glu uptake in the generation of IOS we applied DHK (300 µM), the specific inhibitor of the glial glutamate transporter EAAT2. In the first 5 min of application, DHK enhanced PS amplitude (by 41±19% of control, N = 7, Figure 4C and D), but decreased the summa IOS amplitude (by 42±4% of control) especially in the *str. radiatum* (Figure 4C). Interestingly it also decreased the fEPSP slope (by 39±5% of control). Similarly to the effect of picrotoxin, IOS more closely followed the changes of the fEPSP slope than the PS amplitude. The increased PS amplitude can be explained by the elevated extracellular glutamate concentration [Glu]_o_ [46], [47] that results in an increased firing rate [48] and facilitated response of the neurons to the stimulation [44].

In the next 10 min of drug application all signals decreased (PS amplitude, fEPSP slope and summa IOS amplitude by 27±7%, 58±3% and 59±6% of control, respectively; N = 6). Components resulting in decreased electrophysiological signals could be: *i)* neuronal Glu receptor desensitization [49], [50] due to the increased extracellular [Glu]_o_ [51]; *ii)* partial inactivation of voltage activated sodium channels [52]; *iii)* decreased driving force for Glu-mediated currents [53] and *iv)* long term changes in the baseline swelling of neurons [54], [55]. To test DHK effect on baseline swelling, resting light intensity (RLI) changes were also calculated. There were only small significant changes at both application points (7±2% of control at 5 minutes after DHK application and 5±2% of control further increase in the next 10 minutes). RLI changes indicate the role of baseline cell swelling, however they are relatively small compared to the DHK-sensitive component of the afferent stimulation evoked IOS changes, indicating that baseline swelling is not responsible alone for DHK effect on IOS. Summa IOS amplitude and fEPSP slope decreased almost identically (14±10%, and 19±3% of control, respectively); therefore the IOS decrease in the second stage of drug application can merely be a consequence of the fEPSP slope decrease. DHK affects the slope and decay of IOS in the same manner at both stages, suggesting that this was a consequence of the amplitude decrease. The effect of DHK was reversible as up to 70% of control of the field response and IOS were regenerated after 15 min washout. Therefore we can exclude the excitotoxic neuronal death.

DHK applied simultaneously with APV and CNQX did not decrease further the IOS amplitude and field response parameters (24±1%, 0±1% and 7±1% of control IOS sum. ampl., PS ampl. and fEPSP slope, respectively, N = 5, Figure 4D), suggesting contribution of non-glutamat*ergic* pathways in IOS generation, such as neuronal activity related ATP-release mediated cell swelling [56].

### Contribution of K^+^/Cl^−^ Cotransporter KCC2 but not of Na^+^/K^+^/Cl^−^ Cotransporter NKCC1 to Afferent Stimulation Evoked IOS in Hippocampal Slices

**Figure 5:**
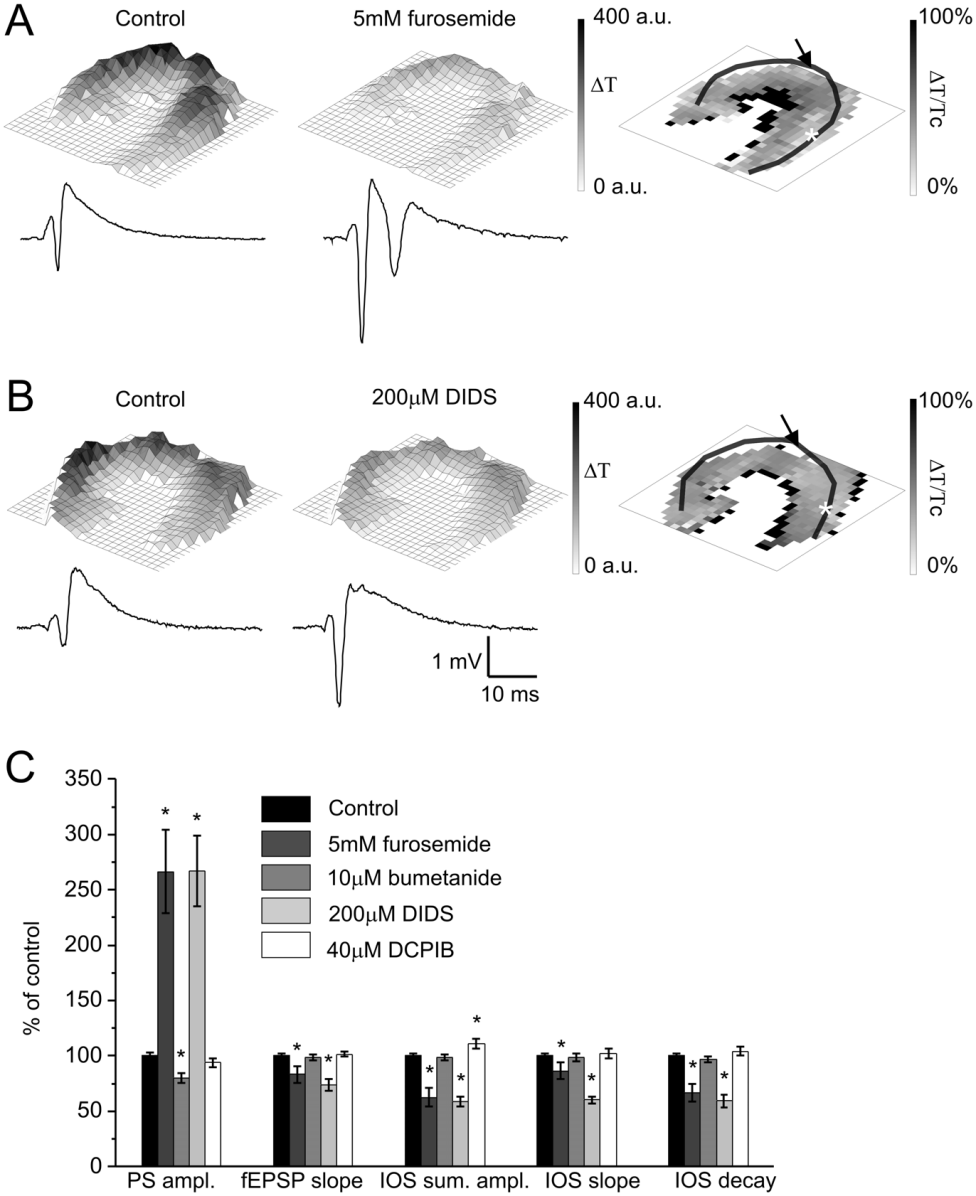
Neuronal K^+^/Cl ^−^ **cotransporter, non-specific Cl** ^−^ **and volume-regulated anion channels contribute to IOS.** **A Left and Middle:** Representative IOS amplitude map and field response curve under control condition and 5 mM furosemide application, respectively. The colorbar indicates the maximum change of the transmittance compared to the resting light intensity. **A Right:** Spatial visualization of the percentage of control changes of IOS signal caused by furosemide application. **B Left and Middle:** Representative IOS amplitude map and field response curve under control condition and 200 µM DIDS application, respectively. The colorbar indicates the maximum change of the transmittance compared to the resting light intensity. **B Right:** Spatial visualization of the percentage of control changes of IOS signal caused by DIDS application. **C:** The effect of 5 mM furosemide, 10 µM bumetanide, 200 µM DIDS and 40 µM DCPIB on the field response and IOS parameters in percentage of control. Asterisks indicate significant changes compared to control (P<0.05 Mann-Whitney U test, N = 5−8). Transparent lines on panel A, B right indicate the pyramidal cell layer. The position of the stimulating and recording electrode are marked by an arrow and asteriks, respectively.

KCC2 transports Cl^−^ and K^+^ ions out of the cells, and is crucial for the maintenance of neuronal intracellular [Cl^−^] [57]. NKCC1 is mainly expressed on astrocytes and transports Na^+^, K^+^ and Cl^−^ ions into the cell [42]. The precise balance between the activities of the two transporters is needed to maintain inhibitory GABA*ergic* signaling in the adult central nervous system [58]. NKCC1 has been suggested to play a decisive role in stimulus evoked IOS due to the dramatic decrease of IOS after application of furosemide [7], [19]. In our experiments furosemide (5 mM), the inhibitor of *both* NKCC1 and KCC2 in the first 5 min of application increased PS amplitude by 226±37% of control while decreased the fEPSP slope by 17±8% of control. The increased neuronal excitability may be due to the impaired GABA_A_ receptor mediated inhibitory function [59] or the reduced K^+^ driving force [60]. In contrast to the PS amplitude, summa IOS amplitude was decreased (by 37±9% of control, N = 8, Figure 5A and C), most prominently in the *str. radiatum* (Figure 5A). It is to note that the IOS decay was more significantly affected (by 33±8% of control) than the slope (by 14±7% of control), suggesting that furosemide is acting on the IOS termination process.

After 10 min, furosemide decreased PS amplitude (by 57±5% of control), fEPSP slope (by 56±5% of control) and summa IOS amplitude (by 73±3% of control, N = 8). The possible explanations of the decreased field potential parameters are that chloride flux alterations diminish vesicular glutamate uptake [61] or stimulus induced extracellular [K^+^] increase is reduced [62]. The remaining IOS signal (27±3% of control) was comparable to that of after inhibition of postsynaptic activation by 20 µM CNQX and 100 µM APV (23±6% of control). This finding is in agreement with previous studies in which furosemide completely blocked postsynaptic activation dependent IOS [7], [45]. After 15 min of washout major fraction of the field response and IOS recovered, therefore neuronal death can be excluded.

Previous studies suggested that action of furosemide on IOS is mediated by the blockade of NKCC1 resulting in reduced glial swelling [7], [63]. However, bumetanide (10 µM), a specific antagonist of NKCC1 did not affect summa IOS amplitude (Figure 5C). The decreased PS amplitude after bumetanide application (20±4% of control, N = 5) may be explained by the reported hyperpolarizing shift of the Cl^−^ reversal potential through inhibition of NKCC1 followed by an increased efficacy of GABA*ergic* inhibition [64]. Since the specific antagonist of NKCC1 did not alter IOS, we concluded the absence of NKCC1 contribution to brief afferent stimulation evoked IOS in the rat hippocampal slice.

### Activation of Non-specific Cl^−^ Channels Enhances, but Activation of Volume-regulated Anion Channel (VRAC) Inhibits Afferent Stimulus Evoked IOS

The inhibition of IOS by furosemide suggested a possible role for Cl^−^ gradient in IOS signals. Therefore we explored the involvement of Cl^−^ channels with different characteristics. Selective VRAC blocker DCPIB (40 µM) did not affect fEPSP slope and PS amplitude while moderately increased summa IOS amplitude (by 11±4% of control, N = 7 Figure 5C). Thus cell swelling evoked by the afferent stimulation protocol activated VRAC. The inhibition of VRAC enhanced IOS, therefore its physiological role is to inhibit cell swelling in afferent stimulation evoked IOS.

In contrast, the general anion channel antagonist DIDS (200 µM) decreased summa IOS amplitude and the fEPSP slope while increased the PS amplitude (by 42±4% of control, 26±6% and 127±32% of control respectively, N = 5, Figure 5B and C). DIDS did not alter the regional distribution of IOS (Figure 5B), indicating that its target have uniform distribution in the slice. DIDS decreased both the slope and decay of IOS, implying that it does not have preference to either IOS generation or termination process. The effect of DIDS on IOS is suggesting that non-specific chloride channels slightly facilitated cell swelling evoked by the stimulus.

In order to explain the field response effect of DIDS, we applied both cell-attached and whole-cell approaches. In the cell-attached approach [Cl^−^] of the investigated cell is maintained at its physiological level, while in the whole-cell current clamp approach the patched cell is exposed to the [Cl^−^] of the pipette solution. Although these experiments were performed on younger (P11−20) animals due to the requirements of the patch clamp technique, there was no difference in the field recording parameters and therefore we suggest that the processes responsible for DIDS effect are matured in these animals. Comparison of the results of cell-attached (increased number of action currents by 128±29% of control) and whole-cell current clamp (increased number of action potentials by 160±67% of control) measurements revealed that the enhanced neuronal activity caused by DIDS is independent of intracellular [Cl^−^].

### Spatial Pattern and the Dynamics of IOS Depends on the Stimulus Type

The effect of DCPIB implied the potential role of cell swelling in afferent stimulation evoked IOS. The question arises whether cell swelling related IOS evoked by a different stimulus could result in a similar spatial pattern and dynamics. To examine the dependency of the IOS characteristics on the stimulation type the spatial pattern and time course of elevated [K^+^]-evoked cell swelling [65], [66], [67] related IOS was compared to the afferent stimulation evoked IOS.

**Figure 6:**
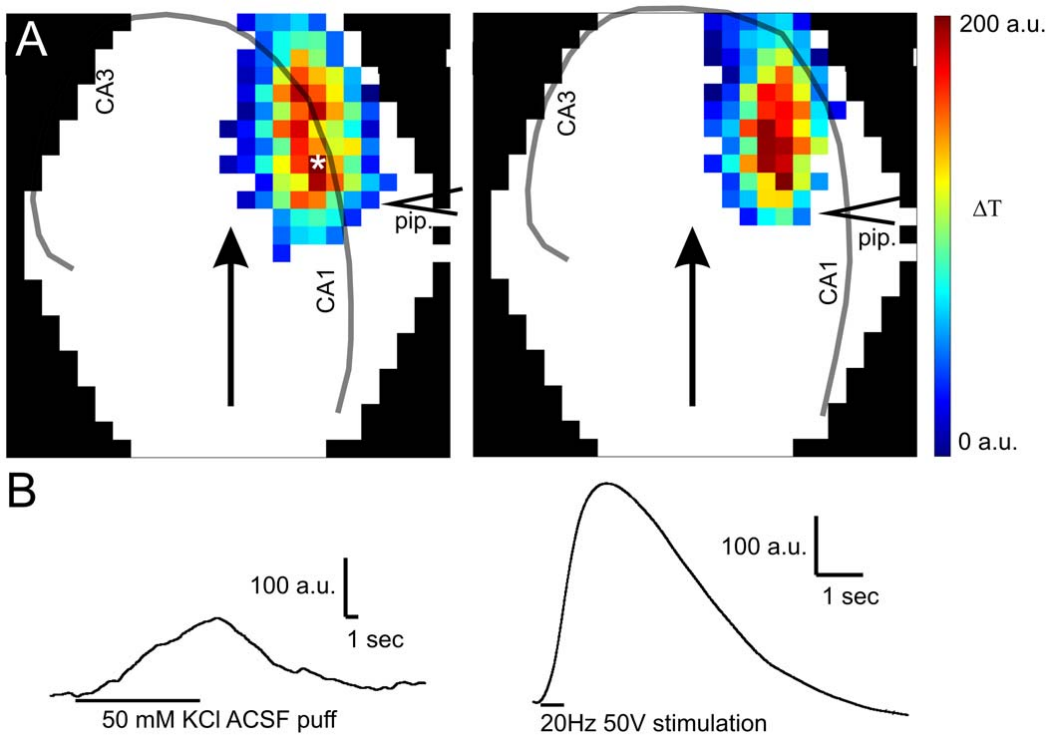
Spatial and temporal patterns of elevated [K^+^]-evoked and afferent stimulation evoked IOS are significantly different. **A:** IOS amplitude maps after application of elevated [K^+^] ACSF ([K^+^] = 50 mM) onto the surface of the slice in the *str. oriens* (**Left**) or in the *str. radiatum* (**Right**) from a patch pipette. The position of the patch pipette (pip.) is indicated by the symbol “<”. Transparent lines indicate the pyramidal layer and the arrow indicates the direction of the buffer flow. The colorbar indicates the maximum change of the transmittance compared to the resting light intensity. **B:** Comparison of the shape of representative IOS traces evoked by elevated [K^+^] (**Left**) and afferent stimulation (**Right**) at the site marked by asterisk in **A**.

IOS was evoked by the local administration of ACSF with [K^+^] = 50 mM from a patch pipette. Normal ACSF administered in the same manner at the same position did not evoke IOS therefore the pressure induced mechanical changes of the slice can be ruled out. Since elevated potassium also depolarizes neurons in addition to inducing cell swelling [68], 50 mM K^+^ ACSF puff was applied in the CA1 *str. oriens* and in the *str. radiatum* to explore whether there are differences in the spatial IOS pattern caused by the different type of neuronal depolarization. IOS was only measurable in front of the puff pipette (Figure 6A), indicating that it represents only the local response of cells to the elevated [K^+^]. Spatial patterns of IOS amplitude were identical regardless of the application site indicating that IOS is related to potassium induced cell swelling and not assigned to neuronal depolarization, since neuronal depolarization would have induced more significant IOS in the pyramidal layer than in the *str. radiatum*.

The time course of the afferent activated and elevated [K^+^]-evoked IOS signals are significantly different (Figure 6B). Both the Schaffer collateral stimulus- and the elevated [K^+^]-evoked signals have positive amplitude and rise rapidly after stimulation onset. Both signals increase further after the termination of its stimulus, but the elevated [K^+^]-evoked signal declines more rapidly than the afferent stimulation evoked IOS.

The differences between the spatial distribution and the dynamics of the elevated [K^+^]-evoked and Schaffer collateral stimulus-evoked IOS suggests that different mechanisms may underlie the signal generation.

## Discussion

In order to better understand the neural excitation related IOS we developed a new paradigm comprising brief afferent stimulation in combination with detection of spatiotemporal progress of IOS at high-frequency by a fast 464-element photodiode-array detector. The new experimental paradigm was applied in acute rat hippocampal slices. Spatiotemporal IOS and local electrophysiological field potentials have been measured simultaneously supplemented with whole cell patch recording when turned to be necessary.

**Figure 7:**
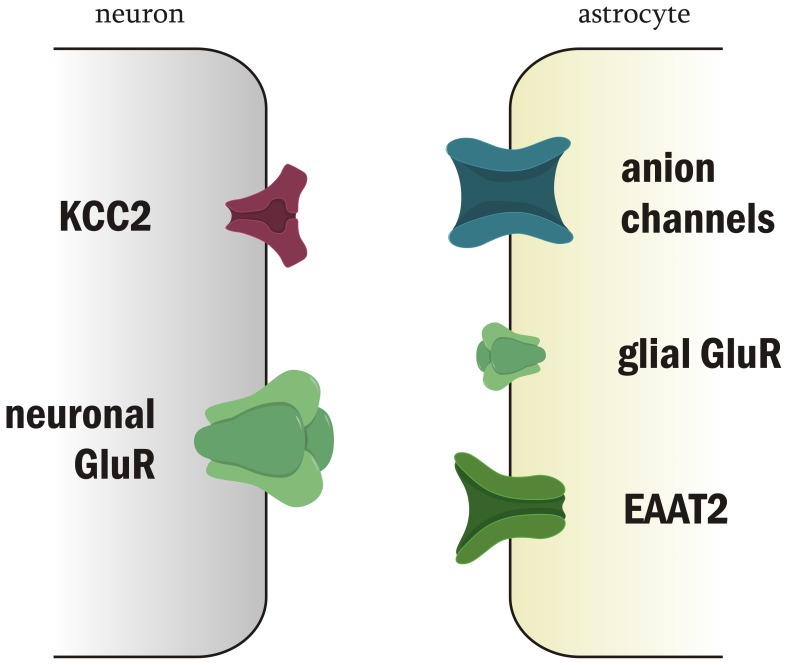
Molecular dissection of processes underlying spatiotemporal IOS generation. Neuronal (left) and glial (right) proteins underlying IOS mechanism are sized in accordance with their contribution to IOS genesis. Neurons are activated first after Schaffer collateral stimulation, followed by neuronal somatic swelling and subsequent swelling of glial cells.

Major findings obtained by this paradigm are summarized as follows: *i*) IOS appears first in the *str. pyramidale*, indicating that neuronal activation precedes IOS signal generation, *ii*) lack of action potentials *via* blockade of voltage-gated Na^+^ channels abolishes IOS generation, *iii*) inhibition of GABA_A_ receptor mediated inhibitory signaling substantially increases IOS, *iv*) IOS evolves through activation of ionotropic Glu receptors, *v*) astrocytes also participate in IOS generation by means of astroglial EAAT2 and Mg^2+^-independent NMDA receptor activation, *vi*) activity of neuronal K^+^/Cl^−^ but not the glial Na^+^/K^+^/Cl^−^ cotransporter contributes to IOS development, connecting neuronal activity to cell swelling, *vii*) VRAC attenuate, while non-specific Cl^−^ channels intensify IOS when activated. Molecular and cellular dissections of processes underlying spatiotemporal IOS generation in hippocampal slices are illustrated in Figure 7.

Small component of IOS in vivo [69], [70], [71] but the almost exclusive source of IOS in the isolated brain studies and in vitro IOS are changes in light scattering and light absorption [7], [16], [17], [18]. Based on the illumination wavelength dependence of IOS [7] we assume that mainly the light scattering component of *in vitro* afferent evoked IOS was detected at 700 nm. Measuring at this wavelength has the advantage of the deeper penetration into the slice and the reduced contribution of the absorption of the residual hemoglobin (usually measured at 600−660 nm [72], [73], [74]) and the intrinsic cytochromes such porphyrins (maximal absorption wavelengths 440 nm [15]). As dendritic beading and changes of mitochondrial architecture are usually coupled by strong activation (i.e. spreading depression, excitotoxicity [2], [13], [14]) we suggest that the main component of our brief afferent activation evoked IOS is cell swelling. This is also supported by previous *in vitro* studies suggesting that afferent evoked IOS attributed to cell swelling [7], [17], [18] because it can be detected at wide range of wavelength with greater effect at longer wavelengths that penetrates deeper [1] and it is sensitive to furosemide and diminution of extracellular [Cl^−^] that inhibit cell swelling [7], [17].

Strong correlation between the local field potential components in the pyramidal layer and local appearance or spatial spreading suggests that IOS reflects the excitability level of the tissue. It is further supported by the 100% elimination of IOS by TTX and its enhancement when neuronal activity was increased by inhibition of inhibitory signaling with GABA_A_ receptor antagonist picrotoxin. We showed that IOS could be inhibited independent of action potential generation by DHK, furosemide and DIDS, revealing active IOS generation processes, including Glu uptake, K^+^/Cl^−^ cotransport and anion channels. In accordance with previous findings [20], [75], the fact that DHK, furosemide and picrotoxin induced IOS changes closely follow fEPSP slope suggests that IOS changes sensitively reflects the changes in excitatory glutamat*ergic* synaptic currents. Applying our *in vitro* IOS paradigm, about 80% of IOS was eliminated by inhibition of ionotropic Glu receptors (AMPA/kainate and NMDA). The majority of previous *in vitro* studies concluded that IOS is a consequence of postsynaptic activation [20], [24], [76] due to the fact that IOS is inhibited by kynureic acid or Ca^2+^-free ACSF [7], [20], [24], [45]. According to these findings we suppose that ionotropic glutamate receptor inhibitors primarily affect IOS by decreasing postsynaptic neuronal activity.

Previous studies describing afferent evoked IOS in hippocampal slices, did not disclose any information regarding the temporal dissection of IOS generation or the spreading of the signal due to the slower imaging device applied in these experiments [1], [7], [24], [45], [63]. PDA enables IOS monitoring at temporal resolution similar to the electrophysiological signal. Utilizing this high-speed acquisition device we showed that neuronal activation precedes IOS in the CA3 and CA1. Surprisingly IOS appears first in the pyramidal layer followed by its initiation in the *str. radiatum* in the CA3 and CA1 regions. This temporal pattern is in clear contrast to a signal with predominantly glial origin. The appearance pattern implies that glial processes related IOS are the consequence of neuronal activation. This view is further supported by IOS increase in the presence of picrotoxin in the pyramidal layer and the more prominent IOS decrease in the *str. radiatum* after application of CNQX. This is also in agreement with the absence of IOS under blockade of neuronal activity.

Neuronal activity induced IOS showed completely different propagation pattern and dynamic in the CA1 and CA3 regions which could be explained by the different type of stimulus received (synaptic contacts vs. antidromic stimulus). The orthodromic and antidromic neuronal activation initiated IOS can be discriminated by the spatial and temporal patterns of the signal. Interestingly, we observed propagation of IOS in the CA1 radiatum toward both ends of the slice along the *str. radiatum*. The spreading velocity of IOS is one order of magnitude slower than the spreading velocity of neuronal activation and one order of magnitude faster that the diffusible mediator related astrocyte Ca^2+^ wave. This may imply the contribution of astroglial syncitium coupled through gap junctions in IOS propagation.

The spreading pattern and temporal resolution of IOS indicated the activation of glial cells that is induced by neuronal activation. Studies on cultured astrocytes [25] showed that glutamate application can induce swelling of astrocytes suggesting their involvement in the glutamat*ergic* neuronal activation dependent IOS generation. Astrocytes have a key role in glutamat*ergic* neurotransmission, since Glu is mainly taken up by astrocytes [77], [78], therefore astroglial Glu uptake is a potential contributor to IOS. In order to clarify the issue, clearance of action potential evoked presynaptically released Glu was blocked by a selective irreversible inhibitor of astroglial Glu uptake. As expected [46], [47], the elevated [Glu]_o_ increased neuronal activity resulting in PS amplitude enhancement, most likely arising from intensified neuronal firing rate [48]. Concurrently, blockade of astroglial Glu uptake decreased summa IOS amplitude indicating that Glu robustly taken up by the astroglial EAAT2 [79] also shapes the *in vitro* apparent IOS. The decrease of summa IOS amplitude can be explained by the blockade of Glu uptake mediated astrocytic cell swelling [25], [26]. The role for astroglia in shaping *in vitro* apparent IOS is further substantiated by the fact that the most prominent IOS decrease to EAAT2 blockade was observed in the *str. radiatum*, a hippocampal area rich in astrocytes [80], [81]. It is worth noting that blood volume changes related *in vivo* IOS signal is also attributed to astrocytic glutamate uptake [27] suggesting the role of astrocytes in both signals.

Furthermore, the additional inhibitory effect of NMDA antagonism while AMPA/kainate receptors were blocked may indicate some added astroglial contribution to the *in vitro* IOS generation by way of Mg^2+^-independent astroglial NMDA receptor activation [42], [43].

As mentioned above, about 20% share of *in vitro* apparent IOS is not due to proper ionotropic glutamat*ergic* activity since it cannot be blocked by AMPA/kainate and NMDA receptor antagonists. In order to explain the residual IOS component we may conjecture an astroglial Glu uptake process arising from presynaptic Glu release [82]. However, simultaneous blockade of the EAAT2 transporter in the presence of NMDA and AMPA/kainate receptor antagonists left the IOS unchanged implying that it is not triggered by Glu release from presynaptic terminals. Thus, the source of remaining IOS could be activation of other pathways by Schaffer stimulation. Neuronal activity-dependent astroglial IOS component evoked by non-vesicular release of ATP from swelling axon may also be a candidate for accounting residual IOS [56], [83], [84].

Afferent stimulation evoked IOS *in vitro* could be blocked by furosemide and lowered extracellular [Cl^−^] [7], [17]. It is proposed that afferent evoked IOS mainly represents astroglial cell swelling by KCl uptake via the furosemide-sensitive NKCC1 [7], [17]. However, furosemide inhibits neuronal KCC2 in addition to astroglial NKCC1 [85], [86] and these cotransporters were found to take part in regulation of neuronal activity [85], [86]. Their blockers such as furosemide and bumetanide also turned out to be anticonvulsant in epilepsy models [62], [87]. In our study furosemide, a non-specific blocker of KCC2 and NKCC1 exhibited a two-phase effect on the field response. Furosemide first increased the PS amplitude likely through the alteration of GABA_A_ mediated Cl^−^ current reversal potential E_GABA_ [88], but decreased fEPSP slope. In the second phase all field response parameters were decreased. These findings may be explained by the decrease of stimulation evoked increase of extracellular [K^+^] [62] known to contribute to the anticonvulsant effect of furosemide [62]. Our finding is in agreement with the findings of Gutschmidt et al. [62], but conflicting with the results of MacVicar and Hochmann [7] who states that furosemide did not change field response amplitude. The differences might arise from different duration of drug application.

In contrast to electrophysiological signal, furosemide decreased IOS amplitude in both phases. Surprisingly, the NKCC1-specific inhibitor bumetanide left the IOS unaffected excluding the primary role for NKCC1 in generating afferent stimulus evoked IOS *in vitro*. This implies that the extracellular [K^+^] elevation evoked by neural activity may contribute to IOS generation, in particular when taking into account the K^+^ buffering capacity of astroglial cells [62]. Also, direct contribution of KCC2, expressed mainly by neuronal cells [89] to IOS cannot be excluded. It is worth mentioning that according to Gutschmidt et al. [62] the antiepileptic action of furosemide also lacks the contribution of NKCC1. Moreover, inhibition of NKCC1 by bumetanide decreased PS amplitude and IOS remained unchanged while inhibition of both NKCC1 and KCC2 by furosemide increased PS amplitude and decreased IOS. Qualitatively distinguishable effects of inhibition of NKCC1 only (by bumetanide) and NKCC1 with KCC2 (by furosemide) conclusively suggest that the impact of furosemide resides in the inhibition of KCC2. Importantly, inhibition of both cation/Cl^−^ cotransporters decoupled IOS from the neuronal activity.

Increasing evidence suggest that cells regulate their volume through cell swelling activated VRAC that generates regulatory volume decrease [90], [91]. It has been shown that VRAC outwardly rectifies Cl^−^ and is also permeable for water and organic anions such as taurine [92], [93]. VRAC-mediated ATP release has been reported after electrical stimulation evoked IOS [83], suggesting the contribution of VRAC to stimulus evoked IOS. We found that the selective VRAC blocker DCPIB did not affect evoked potential and moderately increased IOS amplitude. In contrast, the general anion channel antagonist DIDS decreased IOS amplitude indicating that non-specific Cl^−^ channels facilitated cell swelling. Unlike IOS, the field response amplitude and neuronal excitability was increased in the presence of DIDS. The enhancement of neural activity also occurred when the intracellular [Cl^−^] was set by patch pipette in whole cell current clamp measurements suggesting that neuronal excitability was increased in a Cl^−^ independent way. Our findings on the effects of regulatory volume decrease by VRAC and swelling performed by non-specific Cl^−^ channels demonstrate the contribution of neuronal activity evoked cell swelling to IOS.

We have shown the significance of three new players (EAAT2, non-specific Cl^−^ channels, VRAC) known to contribute to cell swelling in afferent evoked IOS generation. The question arises whether cell swelling related IOS evoked by a different stimulus could result in a similar spatial pattern and dynamics. We compared the spatial pattern of elevated [K^+^]-evoked IOS to afferent stimulation evoked IOS. Both signals were light transmittance increase suggesting the similarity of the signal generation processes. Elevated extracellular [K^+^] can depolarize neurons [68] therefore could induce IOS by increasing neuronal activation, but in our experiments elevated [K^+^]-evoked IOS did not show any neuronal activity specific spatial pattern, it merely reflected the potassium gradient. The elevated [K^+^]-evoked IOS and afferent stimulation evoked IOS showed clearly different spatial and temporal patterns. Elevated [K^+^]-evoked IOS only shortly increased after the termination of stimulus while afferent stimulation evoked IOS reached its peak long after the cessation of stimulation. The most significant difference is in the decay phase of the signals. Elevated [K^+^]-evoked IOS decays more rapidly than afferent stimulation evoked IOS. The difference in the dynamics of the signals implies that different mechanisms may underlie the two processes. Both signals are thought to represent cell swelling, however the mechanism of cell swelling may be different (i.e. Glu uptake induced or KCl uptake induced).

We propose the following mechanism of afferent evoked IOS generation: first, neurons in the pyramidal layer are activated leading to swelling of their soma, followed by swelling of glial cells in the dendritic region. This is suggested by the facts that IOS first appears in the *str. pyramidale* and also by the TTX- and Glu receptor activation sensitivity of the signal. Glial activity related IOS appears as the consequence of neuronal activation. Neurons induce glial IOS through elevated extracellular [K^+^] concentration by depolarization, by their KCC2 cotransporter and by the release of glutamate that activates EAAT2 and glial Mg^2+^-independent NMDA receptors. Cell swelling induced by Glu or KCl uptake activates VRAC which is responsible for the decay phase of the signal. Components taking part in the IOS generation and the difference between the spatial and temporal IOS pattern of the ortho- and antidromically activated areas imply that IOS reflects the intimate communication between neurons and glial cells. Further investigating the components of afferent evoked IOS by high frequency imaging and further revealing the detailed mechanism of IOS may give us deeper insights into the spatial and temporal mechanisms of neuronal tissue function.

### Conclusions

A new *in vitro* approach was developed to better understand cellular and molecular mechanisms underlying afferent evoked IOS genesis. By applying *in vivo* mimicking afferent stimulation of Schaffer collaterals in combination with high-frequency IOS imaging in hippocampal slices, IOS was found to be directly dependent on neuronal activity as demonstrated by inhibition of major players such as voltage-gated Na^+^ channels, GABA_A_ or postsynaptic AMPA and NMDA receptors. Astroglial EAAT2, cation/Cl^−^ cotransporters and non-specific Cl^−^ channels also associate IOS to neuronal activity, translating transient electric activity to a more lasting and robust *opto-osmotic* signal. It may have relevance in this respect that VRAC only slightly contributes to IOS. As demonstrated in this paper IOS is a complex signal representing complex neuron-to-astroglia signalling. We assume therefore that IOS more genuinely manifests brain (dis)functioning than the electric activity alone. Further studies are needed to make straight comparison of *in vitro* and *in vivo* IOS to broaden our understanding of *in vivo* IOS mechanisms and better assist diagnosis in the future.

## Supporting Information

Figure S1 **Comparison of the effect of different drug on IOS in the sole CA1 and CA3 as well as in the whole slice.** Changes of sum of all IOS parameters calculated for the sole CA1 (black), CA3 (red) and the whole slice (blue) are compared. Except in the case of DHK, the effects of the drugs were not significantly different for the sole CA1 and CA3 when compared to the changes of the IOS for sum of the whole slice.
(TIF)

Video S1 **Temporal pattern of IOS generation.** The video shows the IOS amplitude map starting at the onset of the first stimulus (0.6 ms, 2nd frame) to the onset of the third stimulus (100 ms, 165th frame). The second stimulus is applied at 50 ms (83rd frame). Each pixel corresponds to one photodiode of the PDA detector and covers an approximately 70×70 µm area. The position of the stimulating electrode is marked by an arrow. The colorbar indicates IOS amplitude in arbitrary unit.
(AVI)

Video S2 **Overview of the temporal changes of afferent evoked IOS amplitude.** The video shows the IOS amplitude map starting at the onset of the first stimulus (0 ms, 1st frame) to the end of the experiment (9800 ms, 164th frame). The stimulation lasts for 450 ms (frames #2–7). Each pixel corresponds to one photodiode of the PDA detector and covers an approximately 70×70 µm area. The position of the stimulating electrode is marked by an arrow. The colorbar indicates IOS amplitude in arbitrary unit.
(AVI)

## References

[1] Aitken PG, Fayuk D, Somjen GG, Turner DA (1999) Use of intrinsic optical signals to monitor physiological changes in brain tissue slices. Methods 18: 91–103. 10356339 10.1006/meth.1999.0762

[2] Fayuk D, Aitken PG, Somjen GG, Turner DA (2002) Two different mechanisms underlie reversible, intrinsic optical signals in rat hippocampal slices. J Neurophysiol 87: 1924–1937. 11929912 10.1152/jn.00231.2001

[3] Chen-Bee CH, Agoncillo T, Lay CC, Frostig RD (2010) Intrinsic signal optical imaging of brain function using short stimulus delivery intervals. J Neurosci Methods 187: 171–182. 20079373 10.1016/j.jneumeth.2010.01.009 PMC2832718

[4] Mc Loughlin NP, Blasdel GG (1998) Wavelength-dependent differences between optically determined functional maps from macaque striate cortex. NeuroImage 7: 326–336. 9626673 10.1006/nimg.1998.0329

[5] Rector DM, Poe GR, Kristensen MP, Harper RM (1997) Light scattering changes follow evoked potentials from hippocampal Schaeffer collateral stimulation. J Neurophysiol 78: 1707–1713. 9310454 10.1152/jn.1997.78.3.1707

[6] Zepeda A, Arias C, Sengpiel F (2004) Optical imaging of intrinsic signals: recent developments in the methodology and its applications. J Neurosci Methods 136: 1–21. 15126041 10.1016/j.jneumeth.2004.02.025

[7] MacVicar B, Hochman D (1991) Imaging of synaptically evoked intrinsic optical signals in hippocampal slices. J Neurosci 11: 1458–1469. 1851222 10.1523/JNEUROSCI.11-05-01458.1991 PMC6575307

[8] Pouratian N, Cannestra AF, Martin NA, Toga AW (2002) Intraoperative optical intrinsic signal imaging: a clinical tool for functional brain mapping. Neurosurg Focus 13: e1. 10.3171/foc.2002.13.4.2 15771400

[9] Prakash N, Uhlemann F, Sheth SA, Bookheimer S, Martin N, et al (2009) Current trends in intraoperative optical imaging for functional brain mapping and delineation of lesions of language cortex. NeuroImage 47 Suppl 2T116–126. 18786643 10.1016/j.neuroimage.2008.07.066 PMC2782948

[10] Schwartz TH, Chen LM, Friedman RM, Spencer DD, Roe AW (2004) Intraoperative optical imaging of human face cortical topography: a case study. Neuroreport 15: 1527–1531. 15194889 10.1097/01.wnr.0000131006.59315.2f

[11] Kawauchi S, Sato S, Ooigawa H, Nawashiro H, Ishihara M, et al (2009) Light scattering change precedes loss of cerebral adenosine triphosphate in a rat global ischemic brain model. Neurosci Lett 459: 152–156. 19446006 10.1016/j.neulet.2009.05.014

[12] Haglund MM, Meno JR, Hochman DW, Ngai AC, Winn HR (2008) Correlation of intrinsic optical signal, cerebral blood flow, and evoked potentials during activation of rat somatosensory cortex. J Neurosurg 109: 654–663. 18826352 10.3171/JNS/2008/109/10/0654

[13] Jarvis CR, Lilge L, Vipond GJ, Andrew RD (1999) Interpretation of intrinsic optical signals and calcein fluorescence during acute excitotoxic insult in the hippocampal slice. NeuroImage 10: 357–372. 10493895 10.1006/nimg.1999.0473

[14] Muller M, Somjen GG (1999) Intrinsic optical signals in rat hippocampal slices during hypoxia-induced spreading depression-like depolarization. J Neurophysiol 82: 1818–1831. 10515971 10.1152/jn.1999.82.4.1818

[15] Mane M, Muller M (2012) Temporo-spectral imaging of intrinsic optical signals during hypoxia-induced spreading depression-like depolarization. PloS ONE 7: e43981. 22952835 10.1371/journal.pone.0043981 PMC3430631

[16] Federico P, Borg SG, Salkauskus AG, MacVicar BA (1994) Mapping patterns of neuronal activity and seizure propagation by imaging intrinsic optical signals in the isolated whole brain of the guinea-pig. Neuroscience 58: 461–480. 8170533 10.1016/0306-4522(94)90073-6

[17] Holthoff K, Witte OW (1996) Intrinsic optical signals in rat neocortical slices measured with near-infrared dark-field microscopy reveal changes in extracellular space. J Neurosci 16: 2740–2749. 8786449 10.1523/JNEUROSCI.16-08-02740.1996 PMC6578770

[18] Holthoff K, Witte OW (1998) Intrinsic optical signals in vitro: a tool to measure alterations in extracellular space with two-dimensional resolution. Brain Res Bull 47: 649–655. 10078621 10.1016/s0361-9230(98)00135-x

[19] Andrew RD, Jarvis CR, Obeidat AS (1999) Potential sources of intrinsic optical signals imaged in live brain slices. Methods 18: 185–196, 179. 10.1006/meth.1999.0771 10356350

[20] Cerne R, Haglund MM (2002) Electrophysiological correlates to the intrinsic optical signal in the rat neocortical slice. Neurosci Lett 317: 147–150. 11755261 10.1016/s0304-3940(01)02453-3

[21] Dodt HU, Zieglgänsberger W (1998) Visualization of neuronal form and function in brain slices by infrared videomicroscopy. Histochem J 30: 141–152. 10188923 10.1023/a:1003291218707

[22] Syková E, Vargová L, Kubinová S, Jendelová P, Chvátal A (2003) The relationship between changes in intrinsic optical signals and cell swelling in rat spinal cord slices. NeuroImage 18: 214–230. 12595177 10.1016/s1053-8119(02)00014-9

[23] Kohn A, Metz C, Quibrera M, Tommerdahl MA, Whitsel BL (2000) Functional neocortical microcircuitry demonstrated with intrinsic signal optical imaging in vitro. Neuroscience 95: 51–62. 10619461 10.1016/s0306-4522(99)00385-1

[24] Dodt HU, D’Arcangelo G, Pestel E, Zieglgänsberger W (1996) The spread of excitation in neocortical columns visualized with infrared-darkfield videomicroscopy. Neuroreport 7: 1553–1558. 8904754 10.1097/00001756-199607080-00004

[25] Han BC, Koh SB, Lee EY, Seong YH (2004) Regional difference of glutamate-induced swelling in cultured rat brain astrocytes. Life Sci 76: 573–583. 15556169 10.1016/j.lfs.2004.07.016

[26] Schneider GH, Baethmann A, Kempski O (1992) Mechanisms of glial swelling induced by glutamate. Can J Physiol Pharmacol 70 Suppl: S334–343 10.1139/y92-280 1363532

[27] Gurden H, Uchida N, Mainen ZF (2006) Sensory-evoked intrinsic optical signals in the olfactory bulb are coupled to glutamate release and uptake. Neuron 52: 335–345. 17046695 10.1016/j.neuron.2006.07.022

[28] Lasztóczi B, Antal K, Nyikos L, Emri Z, Kardos J (2004) High-frequency synaptic input contributes to seizure initiation in the low-[Mg^2+^] model of epilepsy. Eur J Neurosci 19: 1361–1372. 15016094 10.1111/j.1460-9568.2004.03231.x

[29] Lasztóczi B, Nyitrai G, Héja L, Kardos J (2009) Synchronization of GABAergic inputs to CA3 pyramidal cells precedes seizure-like event onset in juvenile rat hippocampal slices. J Neurophysiol 102: 2538–2553. 19675286 10.1152/jn.91318.2008

[30] Chang PY, Jackson MB (2003) Interpretation and optimization of absorbance and fluorescence signals from voltage-sensitive dyes. J Membr Biol 196: 105–116. 14724747 10.1007/s00232-003-0629-8

[31] Glykys J, Mody I (2007) The main source of ambient GABA responsible for tonic inhibition in the mouse hippocampus. J Physiol 582: 1163–1178. 17525114 10.1113/jphysiol.2007.134460 PMC2075237

[32] Anderson WW, Collingridge GL (2001) The LTP Program: a data acquisition program for on-line analysis of long-term potentiation and other synaptic events. J Neurosci Methods 108: 71–83. 11459620 10.1016/s0165-0270(01)00374-0

[33] Andersen P, Bliss TVP, Skrede KK (1971) Unit analysis of hippocampal population spikes. Exp Brain Res 13: 208–221. 5123965 10.1007/BF00234086

[34] Andersen P, Blackstad TW, Lömo T (1966) Location and identification of excitatory synapses on hippocampal pyramidal cells. Exp Brain Res 1: 236–248. 5920551 10.1007/BF00234344

[35] Buchheim K, Wessel O, Siegmund H, Schuchmann S, Meierkord H (2005) Processes and components participating in the generation of intrinsic optical signal changes in vitro. Eur J Neurosci 22: 125–132. 16029202 10.1111/j.1460-9568.2005.04203.x

[36] Kuo IY, Wolfle SE, Hill CE (2011) T-type calcium channels and vascular function: the new kid on the block? J Physiol 589: 783–795. 21173074 10.1113/jphysiol.2010.199497 PMC3060357

[37] Barbarosie M, Avoli M (1997) CA3-driven hippocampal-entorhinal loop controls rather than sustains in vitro limbic seizures. J Neurosci 17: 9308–9314. 9364076 10.1523/JNEUROSCI.17-23-09308.1997 PMC6573610

[38] Tzingounis AV, Wadiche JI (2007) Glutamate transporters: confining runaway excitation by shaping synaptic transmission. Nat Rev Neurosci 8: 935–947. 17987031 10.1038/nrn2274

[39] Haas B, Schipke CG, Peters O, Sohl G, Willecke K, et al (2006) Activity-dependent ATP-waves in the mouse neocortex are independent from astrocytic calcium waves. Cereb Cortex 16: 237–246. 15930372 10.1093/cercor/bhi101

[40] Sul JY, Orosz G, Givens RS, Haydon PG (2004) Astrocytic connectivity in the hippocampus. Neuron Glia Biol 1: 3–11. 16575432 10.1017/s1740925x04000031 PMC1420681

[41] Benson DL, Watkins FH, Steward O, Banker G (1994) Characterization of GABAergic neurons in hippocampal cell cultures. J Neurocytol 23: 279–295. 8089704 10.1007/BF01188497

[42] Deitmer JW, Rose CR (2010) Ion changes and signalling in perisynaptic glia. Brain Res Rev 63: 113–129. 19895844 10.1016/j.brainresrev.2009.10.006

[43] Verkhratsky A, Steinhäuser C (2000) Ion channels in glial cells. Brain Res Brain Res Rev 32: 380–412. 10760549 10.1016/s0165-0173(99)00093-4

[44] Mayer ML, Westbrook GL, Guthrie PB (1984) Voltage-dependent block by Mg2+ of NMDA responses in spinal cord neurones. Nature 309: 261–263. 6325946 10.1038/309261a0

[45] Andrew RD, MacVicar BA (1994) Imaging cell volume changes and neuronal excitation in the hippocampal slice. Neuroscience 62: 371–383. 7830884 10.1016/0306-4522(94)90372-7

[46] Massieu L, Morales-Villagran A, Tapia R (1995) Accumulation of extracellular glutamate by inhibition of its uptake is not sufficient for inducing neuronal damage: an in vivo microdialysis study. J Neurochem 64: 2262–2272. 7722511 10.1046/j.1471-4159.1995.64052262.x

[47] Nyitrai G, Kékesi KA, Juhász G (2006) Extracellular level of GABA and Glu: in vivo microdialysis-HPLC measurements. Curr Top Med Chem 6: 935–940. 16787267 10.2174/156802606777323674

[48] White SR, Duffy P, Kalivas PW (1994) Methylenedioxymethamphetamine depresses glutamate-evoked neuronal firing and increases extracellular levels of dopamine and serotonin in the nucleus accumbens in vivo. Neuroscience 62: 41–50. 7816211 10.1016/0306-4522(94)90313-1

[49] Lozovaya N, Melnik S, Tsintsadze T, Grebenyuk S, Kirichok Y, et al (2004) Protective cap over CA1 synapses: extrasynaptic glutamate does not reach the postsynaptic density. Brain Res 1011: 195–205. 15157806 10.1016/j.brainres.2004.03.023

[50] Nyitrai G, Lasztóczi B, Kardos J (2010) Glutamate uptake shapes low-[Mg^2+^] induced epileptiform activity in juvenile rat hippocampal slices. Brain Res 1309: 172–178. 19912995 10.1016/j.brainres.2009.11.007

[51] Palmer MJ, Taschenberger H, Hull C, Tremere L, von Gersdorff H (2003) Synaptic activation of presynaptic glutamate transporter currents in nerve terminals. J Neurosci 23: 4831–4841. 12832505 10.1523/JNEUROSCI.23-12-04831.2003 PMC3586552

[52] Stuhmer W, Conti F, Suzuki H, Wang XD, Noda M, et al (1989) Structural parts involved in activation and inactivation of the sodium channel. Nature 339: 597–603. 2543931 10.1038/339597a0

[53] Grewer C, Gameiro A, Zhang Z, Tao Z, Braams S, et al (2008) Glutamate forward and reverse transport: from molecular mechanism to transporter-mediated release after ischemia. IUBMB Life 60: 609–619. 18543277 10.1002/iub.98 PMC2632779

[54] Choi DW, Maulucci-Gedde M, Kriegstein AR (1987) Glutamate neurotoxicity in cortical cell culture. J Neurosci 7: 357–368. 2880937 10.1523/JNEUROSCI.07-02-00357.1987 PMC6568898

[55] Choi DW, Rothman SM (1990) The role of glutamate neurotoxicity in hypoxic-ischemic neuronal death. Annu Rev Neurosci 13: 171–182. 1970230 10.1146/annurev.ne.13.030190.001131

[56] Fields RD, Ni Y (2010) Nonsynaptic communication through ATP release from volume-activated anion channels in axons. Sci Signal 3: ra73. 20923934 10.1126/scisignal.2001128 PMC5023281

[57] Balena T, Acton BA, Koval D, Woodin MA (2008) Extracellular potassium regulates the chloride reversal potential in cultured hippocampal neurons. Brain Res 1205: 12–20. 18353290 10.1016/j.brainres.2008.02.038

[58] Kahle KT, Staley KJ, Nahed BV, Gamba G, Hebert SC, et al (2008) Roles of the cation-chloride cotransporters in neurological disease. Nat Clin Pract Neurol 4: 490–503. 18769373 10.1038/ncpneuro0883

[59] Korn SJ, Giacchino JL, Chamberlin NL, Dingledine R (1987) Epileptiform burst activity induced by potassium in the hippocampus and its regulation by GABA-mediated inhibition. J Neurophysiol 57: 325–340. 3559679 10.1152/jn.1987.57.1.325

[60] Traynelis SF, Dingledine R (1988) Potassium-induced spontaneous electrographic seizures in the rat hippocampal slice. J Neurophysiol 59: 259–276. 3343603 10.1152/jn.1988.59.1.259

[61] Otis TS (2001) Vesicular glutamate transporters in cognito. Neuron 29: 11–14. 11182077 10.1016/s0896-6273(01)00176-3

[62] Gutschmidt KU, Stenkamp K, Buchheim K, Heinemann U, Meierkord H (1999) Anticonvulsant actions of furosemide in vitro. Neuroscience 91: 1471–1481. 10391452 10.1016/s0306-4522(98)00700-3

[63] Macvicar BA, Feighan D, Brown A, Ransom B (2002) Intrinsic optical signals in the rat optic nerve: Role for K(+) uptake via NKCC1 and swelling of astrocytes. Glia 37: 114–123. 11754210 10.1002/glia.10023

[64] Dzhala VI, Talos DM, Sdrulla DA, Brumback AC, Mathews GC, et al (2005) NKCC1 transporter facilitates seizures in the developing brain. Nat Med 11: 1205–1213. 16227993 10.1038/nm1301

[65] Hansson E, Muyderman H, Leonova J, Allansson L, Sinclair J, et al (2000) Astroglia and glutamate in physiology and pathology: aspects on glutamate transport, glutamate-induced cell swelling and gap-junction communication. Neurochem Int 37: 317–329. 10812217 10.1016/s0197-0186(00)00033-4

[66] Pasantes-Morales H, Schousboe A (1989) Release of taurine from astrocytes during potassium-evoked swelling. Glia 2: 45–50. 2523338 10.1002/glia.440020105

[67] Schousboe A, Pasantes-Morales H (1992) Role of taurine in neural cell volume regulation. Can J Physiol Pharmacol 70: S356–S361. 1295685 10.1139/y92-283

[68] Somjen GG (1979) Extracellular potassium in the mammalian central nervous system. Annu Rev Physiol 41: 159–177. 373587 10.1146/annurev.ph.41.030179.001111

[69] Frostig RD, Lieke EE, Ts’o DY, Grinvald A (1990) Cortical functional architecture and local coupling between neuronal activity and the microcirculation revealed by in vivo high-resolution optical imaging of intrinsic signals. Proc Natl Acad Sci USA 87: 6082–6086. 2117272 10.1073/pnas.87.16.6082 PMC54476

[70] Narayan SM, Santori EM, Blood AJ, Burton JS, Toga AW (1994) Imaging optical reflectance in rodent barrel and forelimb sensory cortex. NeuroImage 1: 181–190. 9343569 10.1006/nimg.1994.1003

[71] Narayan SM, Santori EM, Toga AW (1994) Mapping functional activity in rodent cortex using optical intrinsic signals. Cereb Cortex 4: 195–204. 8038568 10.1093/cercor/4.2.195

[72] Ba AM, Guiou M, Pouratian N, Muthialu A, Rex DE, et al (2002) Multiwavelength optical intrinsic signal imaging of cortical spreading depression. J Neurophysiol 88: 2726–2735. 12424307 10.1152/jn.00729.2001

[73] Devor A, Dunn AK, Andermann ML, Ulbert I, Boas DA, et al (2003) Coupling of total hemoglobin concentration, oxygenation, and neural activity in rat somatosensory cortex. Neuron 39: 353–359. 12873390 10.1016/s0896-6273(03)00403-3

[74] Wankhede M, Agarwal N, Fraga-Silva Ra, deDeugd C, Raizada MK, et al (2010) Spectral imaging reveals microvessel physiology and function from anastomoses to thromboses. J Biomed Opt 15: 011111. 20210437 10.1117/1.3316299 PMC2917463

[75] Das A, Gilbert CD (1995) Long-range horizontal connections and their role in cortical reorganization revealed by optical recording of cat primary visual cortex. Nature 375: 780–784. 7596409 10.1038/375780a0

[76] Holtkamp M, Buchheim K, Elsner M, Matzen J, Weissinger F, et al (2011) Status epilepticus induces increasing neuronal excitability and hypersynchrony as revealed by optical imaging. Neurobiol Dis 43: 220–227. 21440625 10.1016/j.nbd.2011.03.014

[77] Coulter DA, Eid T (2012) Astrocytic regulation of glutamate homeostasis in epilepsy. Glia 60: 1215–1226. 22592998 10.1002/glia.22341 PMC3375386

[78] Ransom BR, Ransom CB (2012) Astrocytes: multitalented stars of the central nervous system. Methods Mol Biol 814: 3–7. 22144296 10.1007/978-1-61779-452-0_1

[79] Héja L, Karacs K, Kardos J (2006) Role for GABA and Glu plasma membrane transporters in the interplay of inhibitory and excitatory neurotransmission. Curr Top Med Chem 6: 989–995. 16787274 10.2174/156802606777323656

[80] Bushong EA, Martone ME, Jones YZ, Ellisman MH (2002) Protoplasmic astrocytes in CA1 stratum radiatum occupy separate anatomical domains. J Neurosci 22: 183–192. 11756501 10.1523/JNEUROSCI.22-01-00183.2002 PMC6757596

[81] Jinno S (2011) Regional and laminar differences in antigen profiles and spatial distributions of astrocytes in the mouse hippocampus, with reference to aging. Neuroscience 180: 41–52. 21320577 10.1016/j.neuroscience.2011.02.013

[82] Wu LJ, Steenland HW, Kim SS, Isiegas C, Abel T, et al (2008) Enhancement of presynaptic glutamate release and persistent inflammatory pain by increasing neuronal cAMP in the anterior cingulate cortex. Mol Pain 4: 40. 18823548 10.1186/1744-8069-4-40 PMC2570662

[83] Fields RD (2011) Imaging single photons and intrinsic optical signals for studies of vesicular and non-vesicular ATP release from axons. Front Neuroanat 5: 32. 21852965 10.3389/fnana.2011.00032 PMC3151621

[84] Fields RD (2011) Nonsynaptic and nonvesicular ATP release from neurons and relevance to neuron-glia signaling. Semin Cell Dev Biol 22: 214–219. 21320624 10.1016/j.semcdb.2011.02.009 PMC3163842

[85] Viitanen T, Ruusuvuori E, Kaila K, Voipio J (2010) The K^+^-Cl^−^ cotransporter KCC2 promotes GABAergic excitation in the mature rat hippocampus. J Physiol 588: 1527–1540. 20211979 10.1113/jphysiol.2009.181826 PMC2876807

[86] Zhu L, Polley N, Mathews GC, Delpire E (2008) NKCC1 and KCC2 prevent hyperexcitability in the mouse hippocampus. Epilepsy Res 79: 201–212. 18394864 10.1016/j.eplepsyres.2008.02.005 PMC2394664

[87] Barbaro NM, Takahashi DK, Baraban SC (2004) A potential role for astrocytes in mediating the antiepileptic actions of furosemide in vitro. Neuroscience 128: 655–663. 15381293 10.1016/j.neuroscience.2004.07.007

[88] Huberfeld G, Wittner L, Clemenceau S, Baulac M, Kaila K, et al (2007) Perturbed chloride homeostasis and GABAergic signaling in human temporal lobe epilepsy. J Neurosci 27: 9866–9873. 17855601 10.1523/JNEUROSCI.2761-07.2007 PMC6672644

[89] Rivera C, Voipio J, Payne Ja, Ruusuvuori E, Lahtinen H, et al (1999) The K^+^/Cl^−^ co-transporter KCC2 renders GABA hyperpolarizing during neuronal maturation. Nature 397: 251–255. 9930699 10.1038/16697

[90] Benfenati V, Caprini M, Nicchia GP, Rossi A, Dovizio M, et al (2009) Carbenoxolone inhibits volume-regulated anion conductance in cultured rat cortical astroglia. Channels 3: 323–336. 19713739 10.4161/chan.3.5.9568

[91] Kimelberg HK, MacVicar BA, Sontheimer H (2006) Anion channels in astrocytes: biophysics, pharmacology, and function. Glia 54: 747–757. 17006903 10.1002/glia.20423 PMC2556042

[92] Eggermont J, Trouet D, Carton I, Nilius B (2001) Cellular function and control of volume-regulated anion channels. Cell Biochem Biophys 35: 263–274. 11894846 10.1385/CBB:35:3:263

[93] Nilius B (2004) Is the volume-regulated anion channel VRAC a “water-permeable” channel? Neurochem Res 29: 3–8. 14992260 10.1023/b:nere.0000010430.23137.be

