# Supplementary figures and images for: Neuronal and Astroglial Correlates Underlying Spatiotemporal Intrinsic Optical Signal in the Rat Hippocampal Slice
Source: PLoS One. 2013 Mar 1;8(3):e57694. doi: 10.1371/journal.pone.0057694 (PMC3585794; doi:10.1371/journal.pone.0057694)

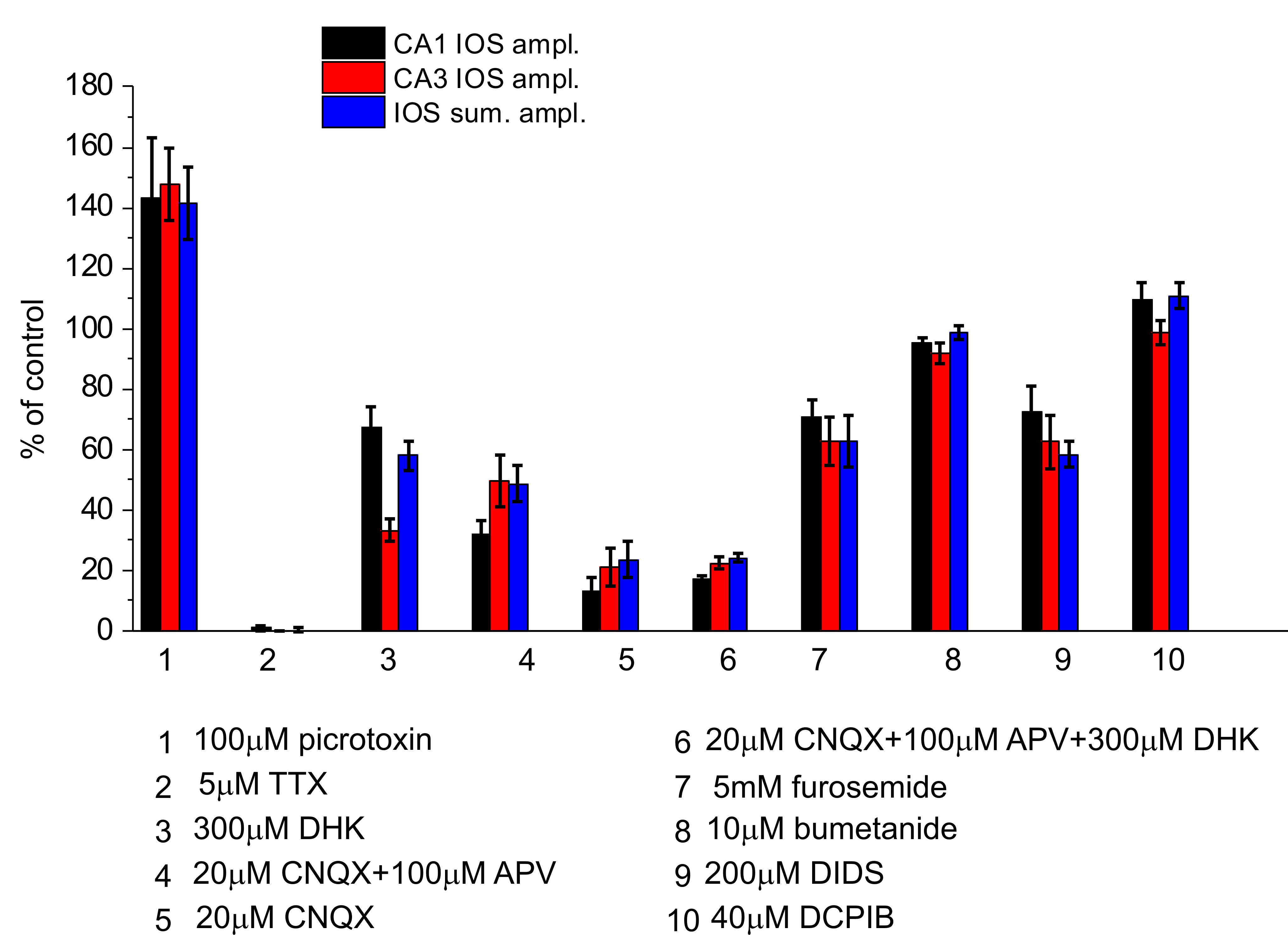

Supplement: Figure S1 — Comparison of the effect of different drug on IOS in the sole CA1 and CA3 as well as in the whole slice. Changes of sum of all IOS parameters calculated for the sole CA1 (black), CA3 (red) and the whole slice (blue) are compared. Except in the case of DHK, the effects of the drugs were not significantly different for the sole CA1 and CA3 when compared to the changes of the IOS for sum of the whole slice. (TIF) [file pone.0057694.s001.tif]
